# In Vitro Activity of the Triazinyl Diazepine Compound FTSD2 Against Drug-Resistant *Mycobacterium tuberculosis* Strains

**DOI:** 10.3390/ph18030360

**Published:** 2025-03-02

**Authors:** Carlos Aranaga, Ruben Varela, Aura Falco, Janny Villa, Leydi M. Moreno, Manuel Causse, Luis Martínez-Martínez

**Affiliations:** 1 Grupo de Investigación en Química y Biotecnología (QUIBIO), Facultad de Ciencias Básicas, Laboratorio de Parasitología y Enfermedades Tropicales, Universidad Santiago de Cali, Santiago de Cali 760035, Colombia; ruben.varela00@usc.edu.co; 2 Departamento de Química Agrícola, Edafología y Microbiología, Universidad de Córdoba, 14071 Córdoba, Spain; luis.martinez.martinez.sspa@juntadeandalucia.es; 3 Grupo de Investigación en Microbiología, Industria y Medio Ambiente (GIMIA), Facultad de Ciencias Básicas, Universidad Santiago de Cali, Santiago de Cali 760035, Colombia; aura.falco@gmail.com; 4 Grupo de Investigaciones Biomédicas, Facultad de Ciencias de la Salud, Corporación Universitaria Remington, Medellín 0500, Colombia; jannyalexander@gmail.com; 5 Grupo de Investigación de Compuestos Heterocíclicos, Departamento de Química, Universidad del Valle, Santiago de Cali 760042, Colombia; leydi.moreno@correounivalle.edu.co; 6 Unidad de Gestión Clínica de Microbiología, Hospital Universitario Reina Sofía, 14004 Córdoba, Spain; manuel.causse.sspa@juntadeandalucia.es; 7 Instituto Maimónides de Investigación Biomédica de Córdoba (IMIBIC), 14004 Córdoba, Spain; 8 Centro de Investigación Biomédica en Red de Enfermedades Infecciosas (CIBERINFECT), Instituto de Salud Carlos III, 28029 Madrid, Spain

**Keywords:** triazinyl diazepine, drug-resistant tuberculosis, antitubercular compounds

## Abstract

**Background/Objectives:** Compounds derived from pyrimido-diazepine have shown selective inhibition of the susceptible *Mycobacterium tuberculosis* strain H37Rv. However, there is a need for studies that evaluate the activity of these compounds against multidrug-resistant strains and clinical isolates. This study aims to evaluate the antitubercular potential of FTSD2 against drug-resistant strains of *M. tuberculosis*. **Methods:** The compound 4-(2,4-diamino-8-(4-methoxyphenyl)-8,9-dihydro-7H-pyrimido[4,5-b][1,4]diazepin-6-yl)-N-(2-(4-(dimethylamino)-6-(4-fluorophenyl)amino-1,3,5-triazin-2-yl)amino)ethyl)benzenesulfonamide (FTSD2) was tested against drug-resistant *M. tuberculosis* strains at minimal inhibitory and bactericidal concentrations (MIC and MBC). Kill curve assays were performed to assess bactericidal activity, and cytotoxicity was evaluated in human monocyte-derived macrophages and the RAW 264.7 murine macrophage cell line. Intracellular death assays, specifically macrophage infection assays, were also conducted to evaluate the effect of FTSD2 on intracellular *M. tuberculosis* growth. **Results:** FTSD2 inhibited the growth of drug-resistant *M. tuberculosis* at MIC and MBC values between 0.5 and 1 mg/L. Kill curve assays demonstrated concentration-dependent bactericidal activity. No cytotoxicity was observed in macrophages at concentrations below 64 mg/L. Additionally, FTSD2 significantly suppressed intracellular *M. tuberculosis* growth after 192 h. FTSD2 did not inhibit the growth of nontuberculous mycobacteria, including *M. avium*, *M. abscessus*, *M. fortuitum*, *M. chelonae*, and *M. smegmatis* at 50 mg/L. **Conclusions:** FTSD2 exhibits strong potential as a leading compound for the development of new antitubercular drugs, with selective activity against *M. tuberculosis* and minimal cytotoxic effects on macrophages. Further studies are needed to explore its mechanisms of action and therapeutic potential.

## 1. Introduction

With significant advances in the diagnosis and treatment of tuberculosis (TB) in recent years, new options have been developed to combat the disease. However, it remains one of the leading causes of death worldwide due to a single infectious agent [1]. The introduction of new drugs, such as bedaquiline [2], delamanid [3], and pretomanid [4], as well as the repositioning of existing drugs, such as fluoroquinolones, linezolid, clofazimine, and streptomycin, have provided new options for improving the treatment of drug-resistant tuberculosis. In response to this situation, the World Health Organization (WHO) has updated treatment regimens for patients with drug-resistant tuberculosis, emphasizing the use of levofloxacin in cases of isoniazid-monoresistant tuberculosis, as well as the combination of levofloxacin or moxifloxacin, bedaquiline, and linezolid in patients with rifampicin-resistant or multidrug-resistant (MDR) tuberculosis [5]. Despite these advances, significant challenges remain. In 2023, the World Health Organization reported over 400,000 cases of drug-resistant tuberculosis, emphasizing the urgent need for novel therapeutic options to address this growing challenge [6].

Bedaquiline is one of the most widely used new bactericidal drugs for treating patients with MDR-TB [7]. It binds to ATP synthase in both replicating and latent mycobacteria [8]. However, mutations in the *atp*E gene, which encodes the C subunit of mycobacterial ATP synthase, have been shown to confer resistance to this drug [9]. Other mutations, such as those associated with the *pep*Q and *mmp*R genes, may be involved in a moderate increase in the minimum inhibitory concentration (MIC) of both bedaquiline and clofazimine [9,10]. Similarly, mutations associated with the *fbi*A, *fbi*C, *ddn*, and *fgd*1 genes are responsible for resistance to delamanid and pretomanid [9], which are alternatives for patients who cannot be treated solely with drugs from group A (Levofloxacin, moxifloxacin, Bedaquiline, and linezolid) and group B (Clofazimine, Cycloserine, and terizidone) in a prolonged treatment regimen. The emerging resistance to these drugs, as well as to repurposed drugs [7,11,12,13,14], and the toxicity of current TB treatment regimens for some patients continue to limit therapeutic options and present significant challenges in TB care [6]. These findings highlight the need to continue searching for new compounds that can cure all patients.

In a recent study, a series of triazinylamino chalcones exhibited activity by inhibiting the in vitro growth of *M. tuberculosis* H37Rv, with minimum inhibitory concentrations (MICs) ranging from 25 to 50 mg/L. Based on these structures, a series of seven triazinylamino-pyrimido[4,5-b][1,4]diazepines was synthesized, showing a 4- to 10-fold reduction in MIC compared to that of the initial triazinylamino chalcones. Among these compounds, FTSD2 (4-(2,4-diamino-8-(4-methoxyphenyl)-8,9-dihydro-7H-pyrimido[4,5-b][1,4]diazepin-6-yl)-N-(2-(4-(dimethylamino)-6-(4-fluorophenyl)amino-1,3,5-triazin-2-yl)amino)ethyl)benzenesulfonamide) was the most active, with MIC values 2 to 4.5 times lower than the other compounds in the series [15].

Additionally, FTSD2 showed no activity against bacteria such as *Staphylococcus aureus*, *Pseudomonas aeruginosa*, *Klebsiella pneumoniae*, and *Escherichia coli*, even at concentrations exceeding 100 mg/L. This selectivity is particularly relevant in the development of new drugs, as antitubercular treatments are typically prolonged and can affect the patient’s normal microbiota [15].

In contrast, various compounds with heterocyclic systems, including triazines or pyrimido[4,5-b][1,4]diazepine structures, have demonstrated anticancer activity, partly due to their ability to inhibit serine/threonine kinases [16,17,18]. *Mycobacterium tuberculosis* possesses two essential serine/threonine kinases for in vitro survival [19], whereas many other bacteria rely on phosphorylation systems mediated by histidine kinases [20]. Although the molecular target of FTSD2 has not been determined, its selective activity against *M. tuberculosis* suggests that it may act through a mechanism different from that of conventional drugs. In this regard, further characterization of its activity will be key to a better understanding of its therapeutic potential.

However, the standard treatment regimen for drug-susceptible tuberculosis has a 99% efficacy rate. Therefore, efforts to control tuberculosis have focused on finding alternative treatments for rifampicin-resistant tuberculosis (RR-TB), multidrug-resistant tuberculosis (MDR-TB), and extensively drug-resistant tuberculosis (XDR-TB). In this study, we determined the in vitro activity of the triazinyl diazepine compound FTSD2 against drug-resistant reference strains of *M. tuberculosis* and clinical isolates to assess its potential as an antitubercular drug [15,21].

## 2. Results

### 2.1. Susceptibility Testing

The MIC of FTSD2 for *M. tuberculosis*, *M. avium*, and rapidly growing mycobacteria (RGM) is shown in Table 1. For *M. tuberculosis* strains and clinical isolates, the MIC ranged from 0.25 to 1 mg/L. In contrast, the MIC for clinical isolates of *M. avium* and RGM was higher than 100 mg/L, which was the maximum concentration tested in the assays. The MBC/MIC ratio for *M. tuberculosis* was 1, indicating that FTSD2 acts as a bactericidal antibiotic.

### 2.2. Dose−Response of M. tuberculosis

**Figure 1:**
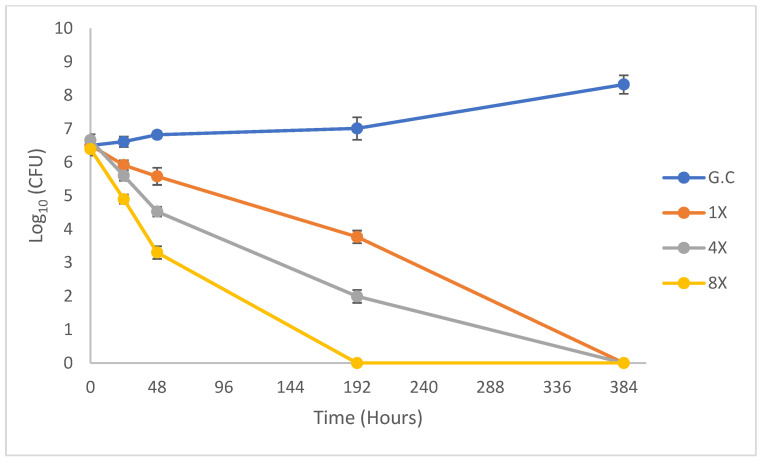
Time-kill curve of *M. tuberculosis* H37Rv. Dose−response of *M. tuberculosis* H37Rv to FTSD2 concentrations of 1× (1 mg/L), 4× (4 mg/L), and 8× (8 mg/L). Data are expressed as the mean and standard deviation.

The time-kill (TK) kinetics of FTSD2 against *M. tuberculosis* H37Rv are presented in Figure 1. The results showed notable bactericidal activity depending on the concentration from 24 h, with a decrease in bacterial concentration of 0.5, 1.1, and 1.5 logarithmic units (Log10) for concentrations of 1× MIC, 4× MIC, and 8× MIC, respectively (Supplementary Material Table S1: Time-kill). At 196 h, no bacterial growth was observed, indicating a reduction of 6.3 Log_10_, while for the same period, the reduction in growth at concentrations of 1× MIC and 4× MIC was 2.9 and 4.7 Log_10_. After 384 h, no bacterial growth was observed at any of the concentrations (Figure 1).

### 2.3. Determination of Cytotoxicity

**Figure 2:**
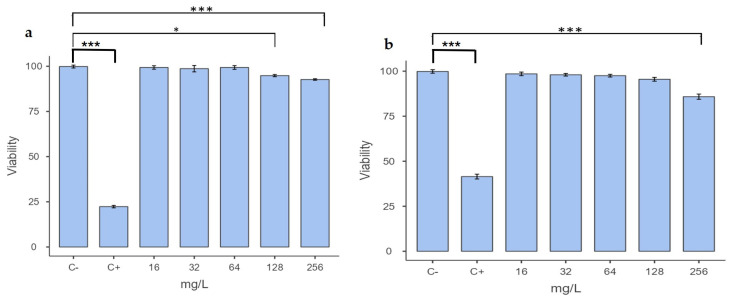
Cytotoxicity of FTSD2 at different concentrations. (**a**) Percentage viability of hMDM cells. (**b**) Percentage viability of RAW 264.7 murine macrophage cells after 48 h of treatment with FTSD2 at different concentrations. C+ (positive control): doxorubicin 2 µg/mL; C− (growth control): 0.1% DMSO. Significant differences were determined using one-way ANOVA followed by Tukey’s post hoc test: * *p* < 0.05, *** *p* < 0.001. Data are expressed as the mean and standard deviation of a representative experiment.

Cell viability assays were performed on human monocyte-derived macrophages (hMDM) and the murine RAW 264.7 macrophage cell line (Figure 2). No significant differences in viability were observed between the growth control and 16, 32, and 64 mg/L concentrations in either cell type. Greater sensitivity to FTSD2 was observed in hMDM, with a statistically significant difference (*p* = 0.015) between the 128 mg/L concentration and the growth control (Figure 2a), while in RAW 264.7 macrophages, significant differences (*p* < 0.001) were only observed at 256 mg/L compared to the growth control (Figure 2b. Supplementary Material Table S2). These results highlight the compound’s high therapeutic index, which combines potent antimycobacterial activity with minimal cytotoxicity.

### 2.4. Macrophage Assay

**Figure 3:**
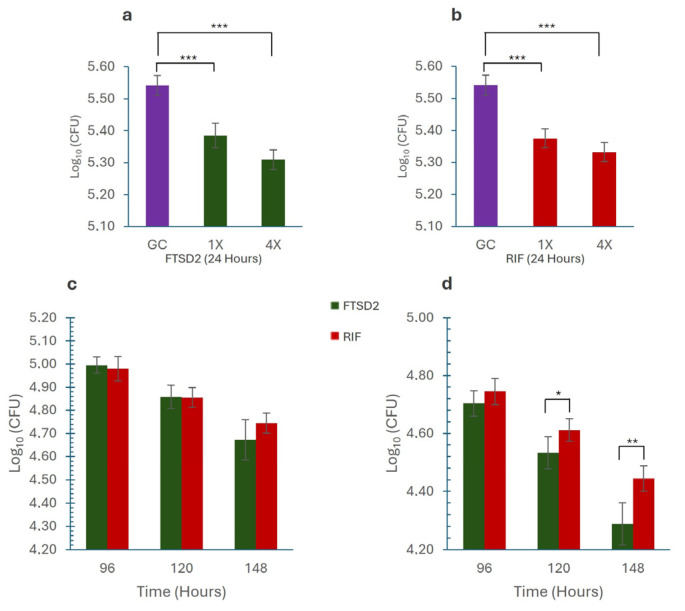
Intracellular Growth Inhibition by *M. tuberculosis*. (**a**) Effect of FTSD2 on the intracellular growth of *M. tuberculosis*. (**b**) Effect of rifampicin on the intracellular growth of *M. tuberculosis*. (**c**) Comparison of the effects of FTSD2 and rifampicin at 1× MIC, showing no significant differences. (**d**) Comparison of the effects of FTSD2 and rifampicin at 4× MIC, showing significant differences after 120 h of exposure. Significant differences were determined using one-way ANOVA followed by Tukey’s post hoc test: * *p* < 0.05, ** *p* < 0.01, *** *p* < 0.001. Data are expressed as the mean and standard deviation of a representative experiment.

The bactericidal activity of FTSD2 was evaluated using rifampicin as a control against *M. tuberculosis* H37Rv in a macrophage infection model, employing concentrations of 1× MIC (1 mg/L FTSD2, 0.2 mg/L RIF) and 4× MIC (4 mg/L FTSD2, 0.8 mg/L RIF). At 24 h, both concentrations significantly reduced bacterial growth compared to that in the control (*p* < 0.001) (Figure 3a,b). This trend persisted throughout the experiment, with more pronounced inhibition observed at 4× than at 1× at all time points, as evidenced by the TK assays. At 1× concentration, FTSD2 exhibited inhibition comparable to that of rifampicin, with no significant differences. However, at 4× concentration, FTSD2 demonstrated greater inhibition, with statistically significant differences at 120 h (*p* = 0.018) and 148 h (*p* = 0.001) compared to rifampicin (Figure 3c,d. Supplementary Material Table S3), suggesting that FTSD2 may sustain its activity for a longer period compared to rifampicin, an aspect of potential relevance for its further development.

## 3. Discussion

The selection of drug-resistant strains currently used against *M. tuberculosis* remains one of the biggest challenges in controlling TB. In 2022 alone, 410,000 cases of TB caused by drug-resistant strains were reported, of which 175,650 patients began treatment [6]. To achieve therapeutic success in managing this resistance, it is essential to have a more significant number of antimicrobials that increase efficacy and reduce toxicity in patients [22]. In this context, we evaluated the potential of a triazinyloxy-pyrimido[4,5-b][1,4]diazepine compound (FTSD2) to inhibit the growth of drug-resistant *M. tuberculosis* strains, including clinical isolates.

The results showed that the minimum inhibitory concentration (MIC) for resistant strains was similar to that observed for susceptible strains H37Ra and H37Rv, with values ranging from 0.25 to 1 mg/L (Table 1). These MICs are comparable to those of several clinically used drugs, such as the aminoglycoside amikacin (1 mg/L), fluoroquinolones ciprofloxacin, ofloxacin, and levofloxacin (0.5–1 mg/L) [23,24], linezolid (0.5–1 mg/L) [25], and the first-line drug ethambutol (1–5 mg/L) [23]. However, FTSD2 MICs were lower than those of capreomycin (10 mg/L) or streptomycin (8 mg/L) [23], suggesting a promising antimicrobial activity profile.

The absence of cross-resistance with other drugs, such as rifampicin, isoniazid, and ethionamide, suggests that FTSD2 acts via a different mechanism of action. Additionally, the MBC was equivalent to the MIC for all tested strains, indicating that FTSD2 exhibits strong bactericidal activity. A previous in vitro study [24], reported that the MIC values of antitubercular drugs such as rifampicin, isoniazid, and levofloxacin were lower than those obtained for the MBC [24]. These results highlight FTSD2 *potential* to eliminate the bacillus in vitro, which could be of interest when considering its combination with other drugs. Additionally, FTSD2 showed no antibacterial activity against nontuberculous mycobacteria, such as *M. avium*, *M. abscessus*, *M. fortuitum*, *M. chelonae*, and *M. smegmatis*, confirming its in vitro antituberculosis specificity.

The in vitro time-kill evaluation showed that FTSD2 exerts a concentration-dependent bactericidal effect, achieving sterilizing activity at 192 h at a concentration of 8 mg/L (8×). This behavior is comparable to that of first-line antitubercular drugs, such as isoniazid, rifampicin, and ethambutol, which have shown concentration-dependent bactericidal effects in previous studies [26,27,28]. However, only rifampicin showed sterilizing activity at 144 h at a concentration of 2 mg/L (8× the drug’s MIC) [27], as observed in this study with FTSD2. Further studies are necessary to determine whether there is synergy between FTSD2 and other antitubercular drugs.

Cytotoxicity assays showed that FTSD2 did not induce toxicity in macrophages at concentrations below 64 mg/L. Higher concentrations (128 and 256 mg/L) were tested to assess the compound’s safety margin and determine the concentration threshold at which cytotoxic effects begin to appear. These assays demonstrated that the cell viability of hMDM and RAW 264.7 macrophages treated with FTSD2 remained above 94% after 48 h of exposure to a concentration of 256 mg/L. Previous studies, such as those reported in [29], revealed that the viability percentage of J774.A1 macrophages treated with rifampicin at a concentration of 10 mg/L was 88% [29]. This suggests that FTSD2 has a competitive advantage in terms of cell safety, especially considering its low toxicity in human and murine macrophages.

Since *M. tuberculosis* proliferates inside macrophages, developing drugs capable of eliminating intracellular mycobacteria is crucial for improving treatment outcomes [29]. FTSD2 was found to exhibit bactericidal activity in a macrophage model, showing prolonged inhibition of intracellular bacterial growth, comparable to that of rifampicin. Additionally, it was found that increasing the drug concentration resulted in a marked reduction in bacterial recovery, indicating greater penetration of the drug into macrophages [30]. The findings of this study suggest that FTSD2 could be integrated into future therapeutic regimens, particularly for multidrug-resistant tuberculosis.

While this study focuses on the in vitro evaluation of FTSD2, it lays the groundwork for future research, particularly in assessing its pharmacokinetics and pharmacodynamics in animal models to further evaluate its efficacy and safety against drug-susceptible and drug-resistant TB and its effectiveness in combination with other antitubercular drugs. Although no pharmacokinetic or animal toxicity studies have been conducted to date, future investigations are necessary to determine the compound’s pharmacokinetic profile, toxicity, and potential in vivo efficacy against drug-resistant tuberculosis. Additionally, it would be interesting to explore the exact mechanism of action of FTSD2 and its potential interactions with emerging resistant mutations, such as those associated with bedaquiline and delamanid. Ultimately, clinical trials are required to confirm its efficacy and safety in humans.

## 4. Materials and Methods

### 4.1. Strains

Seven drug-resistant clinical isolates of *Mycobacterium tuberculosis* were selected from the collection of the Reina Sofía University Hospital in Córdoba, Spain (three resistant to isoniazid, two resistant to isoniazid and rifampicin, and two resistant to isoniazid, rifampicin, and ethionamide), as well as the *M. tuberculosis* strains ATCC 35820, ATCC 35822, ATCC 35837, H37Rv, and H37Ra. Additionally, clinical isolates of *M. avium*, *M. abscessus*, *M. chelonae*, *M. fortuitum*, and the strain *M. smegmatis* mc^2^ 155 were included (Supplementary Material Table S4). Before starting the assays, all strains and clinical isolates were reactivated in Löwenstein−Jensen slant cultures for 3 to 4 weeks at 35 ± 1 °C for *M. tuberculosis* and *M. avium*, while the rapidly growing mycobacteria were incubated at 30 °C for 3 to 5 days.

### 4.2. Triaminopyrimidine-Diazepane Compounds

**Figure 4:**
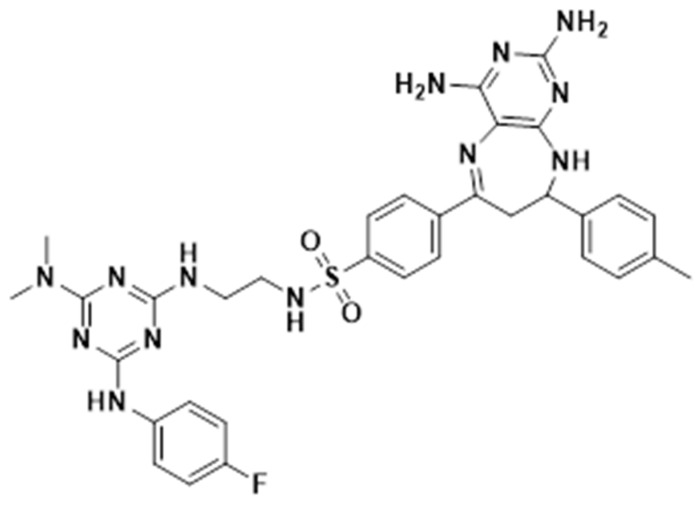
4-(2,4-diamino-8-(p-tolyl)-8,9-dihydro-7H-pyrimido[4,5-b][1,4]diazepin-6-yl)-N-(2-((4-(dimethylamino)-6-((4-fluorophenyl)amino)-1,3,5-triazin-2-yl)amino)ethyl)benzenesulfonamide (FTSD2).

The synthesis of 4-(2,4-diamino-8-(p-tolyl)-8,9-dihydro-7H-pyrimido[4,5-b][1,4]diazepin-6-yl)-N-(2-((4-(dimethylamino)-6-((4-fluorophenyl)amino)-1,3,5-triazin-2-yl)amino)ethyl)benzenesulfonamide (Figure 4) was reported in a previous study [15]. The methodology employed is briefly described as follows: Initially, successive nucleophilic substitution reactions of 2,4,6-trichloro-1,3,5-triazine with 4-fluoroaniline, dimethylamine, and ethylenediamine were carried out, respectively, to obtain N2-(2-aminoethyl)-N4-(4-fluorophenyl)-N6,N6-dimethyl-1,3,5-triazine-2,4,6-triamine. This trisubstituted triazine was then reacted with 4-acetylbenzenesulfonyl chloride in the presence of triethylamine to obtain 4-acetyl-N-(2-((4-(dimethylamino)-6-((4-fluorophenyl)amino)-1,3,5-triazin-2-yl)amino)ethyl)benzenesulfonamide. Subsequently, using this carbonyl precursor as starting material, (E)-N-(2-((4-(dimethylamino)-6-((4-fluorophenyl)amino)-1,3,5-triazin-2-yl)amino)ethyl)-4-(3-(p-tolyl)acryloyl)benzenesulfonamide was obtained by Claisen–Schmidt condensation reactions with 4-methylbenzaldehyde in a basic medium. Finally, diazepine 1 was obtained by reacting (E)-N-(2-((4-(dimethylamino)-6-((4-fluorophenyl)amino)-1,3,5-triazin-2-yl)amino)ethyl)-4-(3-(p-tolyl)acryloyl)benzenesulfonamide with an excess of 2,4,5,6-tetraaminopyrimidine dihydrochloride and BF3·OEt2 in methanol under reflux.

### 4.3. Preparation of Stock Solutions

Due to the moderate aqueous solubility of FTSD2, DMSO was used as a solvent to prepare stock solutions. Stock solutions of FTSD2 and rifampicin were prepared in 100% DMSO at a concentration of 10 mg/mL. Before use in experimental assays, these solutions were diluted in the corresponding media to ensure that the final DMSO concentration in all tests did not exceed 0.1% (*v*/*v*). To eliminate any potential solvent-related interference, drug-free media were supplemented with 0.1% DMSO as a control.

### 4.4. Determination of Minimum Inhibitory Concentration (MIC) and Minimum Bactericidal Concentration (MBC)

The MIC and MBC were determined using the EUCAST broth microdilution method for the *Mycobacterium tuberculosis* complex [31]. Briefly, drug concentrations ranging from 8 mg/L to 0.015 mg/L were prepared in 96-well polystyrene U-bottom plates containing Middlebrook 7H9 medium supplemented with 10% Middlebrook OADC. As a growth control, 100 µL of drug-free medium containing 0.1% DMSO was added. Approximately 5–7 colonies from the Löwenstein−Jensen medium were vortexed in tubes with 3 mm glass beads (Merck, Darmstadt, Germany) and resuspended in 7 mL of distilled water. After 20 min of settling, the supernatant was adjusted to a McFarland standard of 0.5 (1.5 × 10^8^ CFU/mL) and diluted 1:50 to create an inoculum. A total of 100 µL of inoculum was added to each well. Plates were incubated at 35 ± 1 °C for 21 days for slow-growing mycobacteria and 5 days for rapidly growing species. The MIC was defined as the lowest concentration with no visible growth.

For MBC, 50 µL from wells without visible growth were plated on 7H10 medium supplemented with OADC. The plates were incubated at 35 ± 1 °C for 21 days. MBC was defined as the lowest drug concentration that reduced the initial inoculum by 99.9%. All experiments were performed in triplicate.

All experiments were performed in three independent biological replicates, each conducted in technical triplicates.

### 4.5. Kill Rate Determination

The killing rate of *M. tuberculosis* H37Rv exposed to FTSD2 was determined using a time-kill assay. A bacterial suspension adjusted to 0.5 McFarland units was prepared as described in the previous section. A total of 0.2 mL of this solution was added to flasks containing 9.8 mL of culture medium supplemented with the compound at concentrations of 1×, 2×, 4×, and 8× MIC, and a drug-free growth control. Serial dilutions (1:10) were performed for each condition up to a 10^−6^ dilution, and 10 µL aliquots were plated in triplicate on Petri dishes containing 7H10 agar medium supplemented with OADC at 0, 24, 48, 192, and 384 h. The plates were incubated at 36 ± 1 °C for 21 days, and colony counts were performed on dilutions where between 30 and 300 colonies were observed.

Time-kill assays were conducted in two independent experiments, each performed in triplicate.

### 4.6. Cytotoxicity Assay in Macrophages

The cytotoxicity of the FTSD2 compound was evaluated by its ability to eliminate murine RAW 264.7 macrophage cell lines and human monocyte-derived macrophages (hMDMs), using the macrophage-to-myofibroblast transition (MMT) method and the MTT colorimetric assay (3-(4,5-Dimethylthiazol-2-yl)-2,5-diphenyltetrazolium bromide), as described by [32].

#### 4.6.1. MMT Assay

hMDMs were obtained from 50 mL of defibrinated blood from healthy donors, following a density gradient cell separation protocol. Blood was mixed at a 1:1 ratio with calcium- and magnesium-free DPBS (Dulbecco’s Phosphate Buffered Saline). The mixture was then centrifuged at 374× *g* for 20 min at 37 °C using Ficoll-Hypaque 1077 at a 1:3 ratio (blood) to separate the mononuclear cells. The obtained mononuclear cells were washed twice with DPBS and centrifuged again at 15.815× *g* for 10 min. Finally, the cells were resuspended in Dulbecco’s Modified Eagle Medium (DMEM) supplemented with 10% autologous serum, and the concentration was adjusted to 0.3 × 10^6^ cells/mL. The cells were incubated at 37 °C and 5% CO_2_ for 72 h in 24-well plates to promote differentiation into macrophages.

#### 4.6.2. MTT Assay

The MTT assay was carried out in 96-well plates. Both hMDMs and murine RAW 264.7 macrophages were adjusted to a concentration of 2.5 × 10^4^ cells/mL in a supplemented DMEM medium. A total of 180 µL of the cell suspension was added to the wells, followed by 20 µL of FTSD2, to obtain concentrations ranging from 0.5 to 512 times the MIC (0.5 to 512 mg/L). Drug-free medium was used as a growth control. Doxorubicin was used as a positive cytotoxicity control because of its ability to eliminate both tumor and non-tumor cells. The plates were incubated for 48 h at 37 °C and 5% CO_2_. After incubation, the supernatant was removed from each well and the cells were washed with PBS. Fresh medium containing 0.5 mg/mL MTT was then added, and the cells were incubated for an additional 4 h at 37 °C. After incubation, the supernatant was removed, and 100 µL of pure DMSO was added to dissolve the formed crystals, and absorbance was measured at 540 nm using a spectrophotometer. The experiments were performed in triplicate, and statistical significance was determined using one-way ANOVA followed by Tukey’s post hoc test.

Cell viability was calculated using MTT reduction, and cytotoxicity was defined as a decrease in cell viability compared to that of untreated cells. The formula used was: (%) viability inhibition = 1 − [(OD_540nm_ treated cells/OD_540nm_ growth control) × 100], where the absorbance of the growth control represents 100% viability.

Cytotoxicity assays were performed in two independent experiments, each conducted in technical triplicates.

### 4.7. Macrophage Assay

The intracellular activity of FTSD2 was assessed using a macrophage infection model as previously described by Khara et al. (2016) [33] with slight modifications. Briefly, RAW 264.7 cells were seeded at 2.5 × 10^4^ cells per well in 96-well plates and incubated for 24 h to allow adherence. Prior to infection, *M. tuberculosis* H37Rv cultures were washed twice with PBS, resuspended in DMEM, and added at a final concentration of 3 × 10^5^ cfu per well to achieve an moi of 10:1. After a 4-h incubation at 37 °C and 5% CO_2_, extracellular bacteria were removed by washing with DMEM. Cells were treated with FTSD2 or rifampicin at 1× and 4× MIC. Macrophages were lysed using sterile water for 30 min at different time points (0, 24, 48, 72, 96, 120, and 148 h), and bacterial viability was assessed by plating on 7H10 agar plates. The experiments were performed in triplicate, and statistical significance was determined using one-way ANOVA followed by Tukey’s post hoc test. The results are expressed as log CFU/mL.

Intracellular bacterial growth inhibition assays were performed in two independent experiments, each conducted in technical triplicates.

## Figures and Tables

**Table 1:** MICs of FTSD2 against *Mycobacterium tuberculosis* Strains and Nontuberculous Mycobacteria.

*M. tuberculosis* Strains	Number of Strains	MIC Range (mg/L)	MBC/MIC Ratio
H37Rv	1	1	1
H37Ra	1	1	1
ATCC 35822	1	0.5	1
ATCC 35820	1	1	1
ATCC 35837	1	1	1
**Clinical Isolates**			
Isoniazid-Resistant	3	0.5–1	1
Isoniazid and Rifampicin-Resistant	2	0.25–0.5	1
Isoniazid, Rifampicin, and Ethionamide-Resistant	2	0.5–1	1
**Nontuberculous Mycobacteria**			
*M. avium complex*	2	>50	-
*M. abscessus*	3	>50	-
*M. chelonae*	2	>50	-
*M. fortuitum*	5	>50	-
*M. smegmatis mc^2^155*	1	>50	-

## Data Availability

Data is contained within the article and supplementary material.

## Supplementary Materials

The following supporting information can be downloaded at: https://www.mdpi.com/article/10.3390/ph18030360/s1. Table S1: Time-kill; Table S2: Cytotocicity; Table S3: Macrophage Assay; Table S4: MIC.

## Abbreviations

The following abbreviations are used in this manuscript:

MIC	Minimum inhibitory concentration
MBC	Minimum bactericidal concentration
TB	Tuberculosis
MDR-TB	Multidrug-resistant tuberculosis
hMDMs	Human monocyte-derived macrophages
MMT	Macrophage-to-myofibroblast transition
DPBS	Dulbecco’s phosphate buffered saline
DMSO	Dimethyl sulfoxide
